# Nursing Interventions to Empower Family Caregivers to Manage the Risk of Falling in Older Adults: A Scoping Review

**DOI:** 10.3390/ijerph21030246

**Published:** 2024-02-21

**Authors:** Ana Silva Almeida, Ana Paguia, Ana Paula Neves

**Affiliations:** 1 Setúbal Hospital Center E.P.E., 2910-446 Setúbal, Portugal; 2 Nursing School of Lisbon, 1900-160 Lisbon, Portugal; ana.neves@esel.pt; 3 Sado Family Healthcare Unit, 2910-363 Setúbal, Portugal; ana_paguia@sapo.pt

**Keywords:** accidental falls, older adults, nurse, home environment

## Abstract

Falls pose a significant risk to older adults, resulting in injuries and declining quality of life. The psychological impact, particularly the fear of falling, impairs their well-being. This pervasive fear affects daily activities, leading to self-imposed limitations and reduced engagement. This review aimed to identify nursing interventions to empower family caregivers to manage the risk of falling in older adults. A scoping review was developed following the JBI framework. We searched the CINAHL, MEDLINE, Nursing & Allied Health Collection, Cochrane Central Register of Controlled Trials, MedicLatina, and Cochrane Database of Systematic Reviews. The findings of this review revealed that out of 460 initially identified records, nine articles met the eligibility criteria and were retained for further in-depth analysis. These articles provided insights into nine distinct categories of nurse interventions: Therapeutic Relationships, Family Involvement, Personalized Care, Health Education, Multifactorial Falls Risk Assessment, Home Modifications, Referral, Transition Between Healthcare Services, and Health Care Consultants. The findings of this review have significant implications for clinical practice, particularly in emphasizing the crucial role of nurses in empowering family caregivers and older adults to manage the risk of falling at home. Healthcare professionals, policymakers, and researchers can benefit from this informative resource to develop strategies and guidelines.

## 1. Introduction

Annually, an estimated 37.3 million falls occur with sufficient severity to prompt individuals to seek healthcare services, and of these, 640,000 have fatal consequences [1]. This phenomenon is responsible for an annual loss of over 38 million years of disability-adjusted life, having a more significant impact on years of life with disability than the combined total of injuries caused by transportation, drowning, burns, and poisoning [1,2].

The fear of falling is a significant concern among older adults, as it can trigger difficulties in performing daily life activities and exacerbate anxiety, leading to reduced or absence of physical activity, thereby increasing the risk of falls [3,4,5]. Vitorino et al. [4] also concluded that risk factors associated with the fear of falling included a higher incidence of falls, female gender, age (greater age, greater fear of falling, significantly above 70 years), and poor self-perceived health status. Sedentary, decreased mobility, balance issues, living alone or with a limited social network, and polypharmacy are also risk factors for the prevalence of the fear of falling in older adults [6]. According to Huang [7], understanding the process of managing the fear of falling from the perspective of older adults is a crucial step in empowering them in fall prevention.

At the family level, falls entail direct costs related to the payment for treatments of associated injuries and indirect costs stemming from the financial loss due to time taken off work to care for their family member, psychological distress, and increased time spent in care [8,9]. These factors can lead to an increase in objective and subjective burden and a decrease in their quality of life. Socially, falls result in economic costs due to increased hospitalization time and expenses related to human resources, materials, and additional diagnostic and therapeutic procedures resulting from increased interventions [1,9].

Most older adults reside in domestic environments, where falls are most prevalent [10,11]. The family unit emerges as a pivotal element that requires empowerment to assist individuals in managing the risk of falls [12,13]. Families are the frontline observers of their daily activities and potential hazards. They are uniquely positioned to identify behavior and environmental factors contributing to falls and implement preventive measures effectively [13]. In addition, addressing the risk and fear of falling within the family context acknowledges the emotional and psychological dimensions of aging. Fear of falling can significantly impact an individual’s quality of life, leading to social withdrawal, decreased mobility, and increased dependency [14,15].

Moreover, engaging family caregivers in fall prevention interventions promotes collaboration, shared responsibility, and mutual support. Empowering family members in fall prevention interventions is crucial due to their pivotal role in the daily lives and care of their relatives [16]. As the primary support network, family members often serve as caregivers and decision makers for older adults. Therefore, equipping them with the necessary knowledge and skills to recognize and mitigate fall risks is paramount.

A study on the individual/caregiver dyad sought to understand how older adults and their caregivers perceive the fear of falling. It concluded that being cautious and careful is perceived by both parties differently. Older adults perceive it as overprotection, while caregivers see it as protective. Given these perspectives, the authors highlight the need to conduct studies on fall prevention and fear of falling interventions centered around the family [5]. Ang et al. [17] also mentioned that caregivers are aware of the risk of falls and are willing to assist their family members in preventing falls at home. For them, healthcare professionals need to involve family caregivers in their interventions and value their concerns regarding their family members’ fear of falling at home because the family plays an irreplaceable role in the lives of older individuals with a fear of falling [3].

Whether in hospital or community settings, nurses are healthcare professionals with significant and comprehensive interactions with older adults. Their presence and active involvement are essential to ensuring that older adults receive quality care, appropriate support, and preventive guidance to promote healthy aging and improved quality of life [18].

Previous reviews have primarily focused on interventions aimed at empowering individuals to prevent falls and interventions targeting professional caregivers [19,20]. However, this review marks the first examination of interventions designed to empower family members and informal caregivers. In light of this observation, this study aims to identify nursing interventions to empower family caregivers to manage the risk of falling in older adults.

## 2. Materials and Methods

A scoping review was chosen to achieve the study’s broad objective. Scoping reviews are well-suited to mapping and synthesizing the existing literature on a wide-ranging topic, making them an ideal choice for our research. To implement a structured and systematic approach to conducting this scoping review, we adhered to the framework established by JBI [21].

This report follows the Preferred Reporting Items for Systematic Reviews and Meta-Analyses extension for Scoping Reviews (PRISMA-ScR) guidelines outlined by Tricco et al. [22].

### 2.1. Defining and Aligning the Objective and Question

The PCC (Population, Concept, and Context) mnemonic was used to construct the research question that guided this review.

What are nursing interventions that empower family caregivers to manage the risk of falling in older adults?

### 2.2. Developing and Aligning the Inclusion Criteria with the Objective and Question

Following the PCC (Population, Concept, and Context) mnemonic, inclusion and exclusion criteria were established and are detailed in Table 1.

### 2.3. Describing the Planned Approach to Evidence Searching, Selection, Data Extraction, and Presentation of the Evidence

The leading researcher (A.S.A.) conducted the searches in electronic databases. Following the search, the researcher identified and eliminated duplicate papers, ensuring that each unique study was included only once in the review.

This researcher selected the studies with a second investigator (A.P.). Data extraction was performed by the primary researcher using a standardized instrument that was pilot tested. The other two researchers validated the data extraction table.

### 2.4. Searching for the Evidence

The search strategy was developed to retrieve relevant papers from electronic databases. We searched the following databases: CINAHL Complete, MEDLINE Complete, Nursing & Allied Health Collection, Comprehensive, Cochrane Central Register of Controlled Trials, MedicLatina, and Cochrane Database of Systematic Reviews.

The search was conducted on 24 June 2023, and we considered sources of evidence published in English, Portuguese, and Spanish.

The MeSH descriptors “accidental falls,” “family,” and “nurses,” in conjunction with the Boolean operator “and” were utilized to identify relevant articles.

The search strategy employed in CINAHL Complete was as follows:

S1: “Accidental falls”

S2: “Family”

S3: “Nurses”

Combined search: S1 and S2 and S3.

### 2.5. Selecting the Evidence

Subsequently, a two-step screening process was implemented to identify relevant papers. Initially, two researchers (A.S.A. and A.P.) independently evaluated the titles and abstracts of all identified papers. Subsequently, they independently conducted full-text assessments of potentially relevant papers to ensure complete consensus on meeting the predefined inclusion and exclusion criteria. Disagreements concerning study eligibility were resolved through constructive discussions, with input from a third researcher (A.P.N.) sought when necessary to achieve a consensus on the final selection of papers for inclusion in the scoping review.

### 2.6. Extracting the Evidence

A data extraction instrument for source details, characteristics, and results extraction was created to facilitate the systematic retrieval of information. This form enabled the extraction of critical data elements from each selected study: authors, publication year, study title, country, research aim, study design, and study findings.

To ensure a comprehensive analysis, the leading researcher conducted multiple readings of each selected paper, meticulously identifying and extracting the specific phrases that represented nursing interventions to empower families responsible for caring for older adults at risk of falling. Subsequently, the final extraction chart underwent a comprehensive review and discussion involving all authors.

### 2.7. Analysis of the Evidence

A data-driven thematic analysis was employed to examine and synthesize the collected data systematically. This analytical approach was guided by the framework developed by Braun and Clarke [23]. The primary objective of this analysis was to identify, categorize, and explore recurring themes and patterns present within the selected studies. The emerging themes were subsequently examined and interpreted within the scoping review’s objectives.

It is important to note that, by the scoping review methodology, a quality assessment or critical appraisal of the included studies was not performed, as it was not aligned with the specific objectives of this review. Instead, the focus was on mapping and synthesizing the available evidence to provide a comprehensive overview of the research related to the topic of interest.

## 3. Results

**Figure 1:**
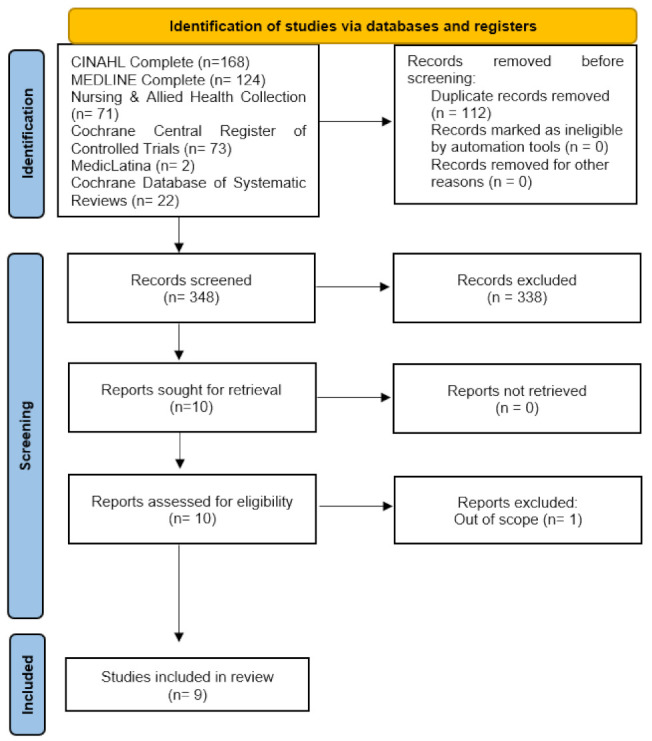
Prisma flow chart.

A total of 460 papers were identified for screening. After eliminating duplicates, 348 titles and abstracts were screened for eligibility, excluding 112 papers. Following an abstract review of 42 articles, ten articles underwent a full-text review, and nine met the eligibility criteria. The flowchart illustrating the screening process is presented in Figure 1.

The included studies spanned publications from 2016 to 2022. Among these studies, five were conducted in the United States of America [24,25,26,27,28], one in Canada [29], one in the United Kingdom [30], one in Brazil [31], and another in Turkey [32] (Table 2).

### 3.1. Therapeutic Relationships

The category of therapeutic relationships in the study highlights the significance of patient–practitioner and family–practitioner relationships in supporting patients’ self-management of falls. Establishing a solid rapport with the patients and their families is crucial in motivating and ensuring exercise adherence [30]. Practitioners recognized the importance of building trust and rapport with patients to enhance adherence to prescriptions.

Furthermore, practitioners also considered cultivating a positive relationship with the patient’s family to be vital. Family caregivers were considered potential facilitators in the patient’s rehabilitation journey [25]. Practitioners emphasized the importance of effective communication with the patient’s family, believing that patients who received consistent support and assistance from family members had better rehabilitation outcomes [30].

### 3.2. Family Involvement

Social networks are pivotal in cultivating secure environments and promoting adherence to fall prevention strategies. In line with Killingback et al. [30], the involvement of family caregivers in the intervention is essential to enhance outcomes, particularly in promoting regular exercise regimens. Kim et al. [24] likewise underscore the significance of involving the families of older adults with managing the risk of falling. Caregivers responsible for individuals in modified homes often seek guidance on maintaining a safe living environment.

### 3.3. Personalized Care

Killingback et al.’s [30] study underscores the essential nature of personalized care in achieving long-term fall prevention. They emphasize that establishing meaningful person-centered goals is crucial for motivating and ensuring the success of fall prevention programs. Montero et al. [29] recommend that healthcare professionals should inquire about the goals, independence, domestic activities, and priorities of families and older adults. Additionally, Olson et al. [25] emphasize the importance of nurses taking into account the valuable insights that family caregivers can provide regarding the home environment and the desired mobility levels for essential activities of their relatives. This can help them design more effective interventions. According to Tan et al. [28], early identification of the needs of the older adult population and matching nursing interventions are crucial to meeting the needs of patients and their families.

### 3.4. Health Education

Several key findings emerged, encompassing various topics related to educating and empowering families that are caring for older adults who fear falling. These results cover various aspects of health education.

One crucial focus should be to raise awareness about falls and their consequences, the importance of identifying signs and symptoms, such as dizziness, loss of consciousness, altered balance or gait, and the fear of falling during an activity, as these factors can heighten the risk of falls [29].

Educating family caregivers on safe techniques for transferring individuals is essential, as highlighted in studies such as Powell-Cope et al. [26]. Research by Olson et al. [25] and Powell-Cope et al. [26] also highlighted the need to educate family caregivers on maintaining mobility and functionality in older adults. Information regarding assistive devices, such as walkers, hygiene chairs, and bathroom grab bars, was also presented by Olson et al. [25]. This information can potentially increase caregivers’ confidence in assisting their family members. Other authors also focus on educating family caregivers to manage the risk of falls and implement fall prevention strategies [25,26,27,29]. To manage the risk of falls, family caregivers can increase safety by learning about environmental factors and architectural modifications [24,25,26,27,29,31,32]. Studies by Montero-Odasso et al. [29], Olson et al. [25], Powell-Cope et al. [26], and Tan et al. [28] underscored the importance of raising awareness of family caregivers regarding the possibility of referral to various healthcare professionals, including family physicians, ophthalmologists, rehabilitation nurses/physiotherapists, and social workers.

Family caregivers should be educated about positive and counterproductive strategies. Positive strategies include engaging in domestic activities together and seeking help from family and friends whenever possible; family caregivers should be educated on responding when a relative experiences a fall [26,27]. This education should include guidance on providing emergency assistance, administering first aid, and assisting the person in getting back up. Caregivers should be equipped with information on how to assess injuries and when to assist the person in getting up rather than calling for emergency help in all cases. Specific situations that are essential to seek immediate assistance can be, for example, when the individual loses consciousness, experiences uncontrolled bleeding, sustains head trauma, or reports significant pain in the hip or other bones, which could indicate a fracture.

Furthermore, Powell-Cope et al. [27] recommend that educational sessions encompass a comprehensive fall prevention and balance assessment approach. In addition to the distribution of brochures, these sessions should also incorporate instructional videos tailored for family caregivers.

### 3.5. Multifactorial Falls Risk Assessment

Olson et al. [25] underscored the pivotal role of nurses in multifactorial fall risk assessment and the planning of fall prevention measures. Within this domain, the information provided by family caregivers assumes a critical role, particularly concerning the history of previous falls, the circumstances surrounding those falls, the recovery process, and the medications administered.

Killingback et al. [30] stress the importance of regular fall risk assessments as well [24,26,31]. Montero et al. [29] support this notion by highlighting the significance of opportunistic health questionnaires. They recommend inquiring older adults and their families about their fall risk during every interaction with healthcare professionals. This assessment should be conducted at least annually, commencing with a straightforward query: “have you experienced any falls in the past 12 months?”. If time allows, two additional questions should follow: “have you encountered balance issues while walking or standing?” and subsequently, “do you fear falling?”. When feasible, it is essential to conduct a comprehensive assessment exploring details such as fall frequency, circumstances, context, severity, and consequences, as well as intrinsic risk factors (physical, psychological, and cognitive conditions), extrinsic factors (home conditions), social aspects, and the older individual’s personal goals, values, beliefs, and priorities.

As noted by Yeni and Yilmaz [32], older adults often maintain independence in their activities of daily living, which are the activities where falls are most likely to occur. This underscores the need for a comprehensive assessment of the individual.

Powell-Cope et al. [27] advocate for a multifactorial assessment that includes the environment. They employed the ‘Check for Safety’ program, primarily designed to enhance home safety. This program offers a practical checklist to identify and address potential hazards within the home environment, spanning areas like the kitchen, bathroom, bedroom, and stairways. The checklist provides valuable recommendations, including removing rugs and decluttering living spaces, improving overall lighting throughout the home, and offering specific solutions for each identified hazard. The program concludes with a set of general tips for fall prevention.

### 3.6. Home Modifications

In the Yeni and Yilmaz [32] study, three home visits were conducted over six months. During the initial visit, participants received an educational session and an informative booklet that covered architectural barriers, risk factors for falls, and other related topics. Subsequent visits reinforced these teachings and assessed the home environment. The most frequently recommended modifications focused on addressing wet or slippery floors. The researchers implemented cost-effective and easily achievable measures, including installing support bars in bathrooms and showers and using non-slip mats in bathing areas to reduce the risk of falls.

Other authors have highlighted the significance of home modifications in reducing the risk of falls. This intervention has emerged as a critical component in fall prevention programs, in line with clinical practice guidelines, as underscored by Kim et al. [24]. Furthermore, Kim et al. [24] identified that the dependence of older adults, their family members’ need for information, and their perception of the risk of falls in their relatives are statistically significant factors associated with the necessity for home modifications.

### 3.7. Referral

Several studies emphasize the importance of referring older adults and family caregivers to various multidisciplinary team members, including family physicians, ophthalmologists, rehabilitation nurses/physiotherapists, and social workers [25,26,28,29]. This ensures they receive comprehensive care and support, addressing their needs and promoting a more effective fall prevention strategy.

Referring caregivers to support groups is another aspect highlighted in the literature [28]. Tan et al. [28] additionally focus on referring individuals to community-based exercise programs, given their benefits in fall prevention.

However, as highlighted by Killingback et al. [30], there is a significant knowledge gap among professionals in community programs. Those who participated in their study mentioned the challenges in obtaining information about community programs and referring clients.

### 3.8. The Transition between Healthcare Services

Killingback et al. [30] highlight a significant gap in terms of transition between Hospital-run exercise-based fall prevention services and community-run exercise programs. This disconnect becomes evident in the perceived unavailability of programs suitable for diverse patient groups and a need for more awareness of community-run programs.

### 3.9. Healthcare Consultants

Kim et al. [24] highlight the importance of nurses collaborating with construction professionals to identify and address structural factors that may lead to falls. This collaborative effort is a critical component of a comprehensive approach to fall prevention, empowering nurses to work alongside construction experts to identify and resolve structural issues that could result in falls.

## 4. Discussion

The results from this review underscore the significance of establishing a therapeutic relationship between healthcare professionals, older adults at risk of falling, and their family caregivers. The findings suggest that a strong therapeutic bond plays a crucial role in providing care. This relationship is not just a formality, but rather an essential bridge that facilitates a deeper understanding of the unique needs and concerns of older adults and their families, ultimately enabling personalized care [33].

The therapeutic relationship involves establishing trust, empathy, and mutual respect [34]. This connection serves as the cornerstone upon which effective care is built. It allows healthcare providers to explore the specific circumstances, preferences, and worries of older adults, as well as the concerns of their family caregivers. By doing so, healthcare professionals gain invaluable insights that guide the tailoring of care plans to address these individualized requirements [33].

The importance of personalized care cannot be overstated, as the fear of falling is a multifaceted issue, and its impact varies from person to person [35,36]. What works for one individual may not necessarily work for another. This is where the therapeutic relationship proves its worth. Through open and honest communication, healthcare professionals can discover the unique factors contributing to an individual’s fear of falling, whether physical limitations, past experiences, or psychological factors.

In the context of fall prevention, the therapeutic relationship acts as a two-way street. Healthcare professionals provide expertise, guidance, and security to older adults and their family caregivers. Simultaneously, they absorb the insights and perspectives of these key stakeholders, enriching their ability to deliver personalized, effective care. It nurtures a collaborative environment where older adults and their families feel heard, valued, and involved in decision making [37,38].

Effective fall prevention strategies include a multifactorial risk assessment and referral to other healthcare professionals [39,40]. As emphasized in various studies, these fundamental elements are essential for developing a comprehensive approach to reducing the risk and fear of falling. The significance of these components lies in their role in achieving a comprehensive and effective approach to fall prevention. A holistic and thorough risk assessment is pivotal in understanding the intricate factors contributing to an individual’s fall susceptibility. This assessment transcends the traditional medical examination and delves into the broader context of an individual’s life. It considers the psychological, social, and environmental factors predisposing someone to falling [40].

Numerous validated fall risk assessment tools exist for multifactorial risk assessments [41]. Extending the use of specialized fall assessment tools to home settings is not only feasible but also highly beneficial. By implementing these tools in home environments, healthcare professionals can gain insights into the unique challenges and risk factors that older adults face in their living spaces. Furthermore, conducting assessments in the home setting allows for a more personalized and targeted approach to fall prevention interventions. This proactive measure empowers older adults and their caregivers to implement practical strategies tailored to their specific needs and circumstances, reducing the risk of falls in the home environment.

A multidimensional view of an older adult’s health and well-being can be obtained by engaging professionals from various disciplines, such as physicians, nurses, physical therapists, and social workers. This interdisciplinary collaboration helps identify intrinsic and extrinsic risk factors that might be missed in a single-discipline assessment. Furthermore, referrals to other healthcare professionals extend this comprehensive approach. After identifying specific risk factors, it is vital to ensure that the individual receives the appropriate interventions and support [42,43].

This review highlights the importance of actively involving family caregivers in the care process to mitigate the risk of falling and enhance the safety of older adults within their homes. A central revelation is establishing an effective care partnership between healthcare professionals and family caregivers [24,30]. This partnership entails the exchange of information, the development of personalized care plans, and collaborative efforts to implement fall prevention strategies. Such collaboration is fundamental for adopting a holistic approach to address the risk of falling in older adults.

Moreover, the review underscores the necessity of educating and empowering family caregivers to assume an active role in fall prevention. This includes training them to identify fall risks, implement safety measures, utilize assistive devices effectively, and address the fear of falling among older adults [44].

Empowering family caregivers with knowledge and skills is pivotal in ensuring the safety of older adults [45]. It is important to explain to families that some strategies can be counterproductive, such as overly close supervision and activity restriction, and should be avoided [17]. Healthcare teams should support the implementation of safe fall prevention strategies, provide explanations, and raise awareness about the risks of specific strategies, such as excessive phone contact with family members [17].

However, it is important to note that providing information alone may not always be sufficient. Educational materials such as brochures, flyers, or books can serve as valuable supplements to reinforce the knowledge gained in educational sessions. These resources act as reference materials that family caregivers can revisit when needed, aiding in the continuous reinforcement of fall prevention strategies [46,47].

Furthermore, in an era where technology continues to evolve and seamlessly integrate into our daily lives, it becomes imperative for healthcare professionals to embrace these advances as tools for enhancing care. Notably, one study highlights educational videos as an exemplary method of leveraging technology for educational purposes [27]. These videos can effectively convey information in an engaging and accessible manner, simplifying complex concepts for better comprehension. Integrating new technologies, such as mobile apps and online platforms, presents an innovative and interactive approach to fall prevention education. These tools offer ongoing support, reminders, and resources for older adults and their family caregivers. Embracing technology enhances the effectiveness of education and aligns with the preferences of a digitally adept society [48]. However, this emphasis on education extends beyond providing general information; it is about equipping older adults and their family caregivers with the knowledge and skills necessary to identify various risk factors and make informed decisions to mitigate these risks proactively.

One key outcome of this review, as highlighted by Killingback et al. [30], is the revelation of a substantial gap in the transition from hospital-based exercise-focused fall prevention services to community-based exercise programs. This gap is evident in the perception of inadequate programs for a broad spectrum of patient groups and a clear need for increased awareness of community-based initiatives. A significant contributing factor to this challenge is the absence of established relationships and collaboration with community-run programs. This prompts questions regarding the responsible parties for supporting older individuals in sustaining their exercise regimens beyond the confines of time-limited hospital interventions.

This raises a critical concern in the healthcare landscape, where the handoff between hospital-based fall prevention services and community-run programs may not be as continuous as necessary. The lack of awareness and accessibility to suitable programs for a diverse patient population further exacerbates this challenge. It highlights the significance of establishing stronger links and collaboration between healthcare providers and community-run programs, bridging the transition gap. Parte superior do formulário

The study by Kim et al. [24] emphasized the growing need for healthcare professionals to collaborate with other professionals, particularly those from the construction industry who serve as healthcare consultants. This interprofessional collaboration has become increasingly significant as healthcare expands its horizons to embrace a more holistic approach to patient care.

The involvement of construction professionals in healthcare consulting highlights the critical need to address structural and environmental factors that can significantly impact individuals’ health and well-being. This partnership aims to create safer and more accessible living environments, especially for older adults and those with specific healthcare needs. One of the critical areas of focus in this collaboration is home modifications and renovations tailored to the unique requirements of individuals, ultimately enhancing their quality of life. These modifications may include installing safety features like grab bars, ramps, and non-slip flooring, as well as improvements to lighting and accessibility. These changes can be vital in preventing accidents, such as falls, and improving overall well-being.

While the results of this review did not specifically identify providing training and consultation to other healthcare professionals, it is crucial to underscore its significance. Training and consultation with healthcare professionals are vital to enhancing their knowledge and skills [49]. By investing in the professional development of healthcare providers, expert health professionals in fall prevention can empower them to be more proactive, adept at identifying potential risks, and skilled in implementing tailored interventions. This benefits the professionals and leads to improved patient outcomes and a healthcare system better equipped to address the complex needs of older adults at risk of falling [49,50].

### Strengths and Limitations

This scoping review has some merits. One of its key strengths lies in its ability to encompass various sources, including empirical studies, literature reviews, and cross-sectional studies, providing a comprehensive overview of the topic. This breadth enables it to identify key themes, trends, and evidence gaps, shedding light on areas that have received substantial research attention and those requiring further investigation.

The review’s findings are valuable for informing decision-making processes. Policymakers, healthcare practitioners, and researchers can use the review’s insights to guide their actions. It is a rich resource that aids in making informed decisions, developing guidelines, and planning effective strategies for fall prevention and fear of falls.

However, the review also presents limitations that should be acknowledged. One of these limitations is the reliance on six databases to identify relevant studies. This approach may overlook studies on the topic that could exist in other databases or sources, potentially limiting the comprehensiveness of the review.

Furthermore, it is essential to consider the language bias, as the review confined the search to papers written in English, Portuguese, and Spanish. By doing so, the review may have inadvertently excluded relevant studies published in other languages. Another limitation is that scoping reviews, by their nature, are more focused on mapping existing literature than on assessing the effectiveness of interventions. Consequently, they may not provide a clear picture of which interventions are the most effective in fall prevention and fear of falling.

This scoping review was performed without any time restrictions. However, the number of reports was low. Therefore, we recommend further investigations into this topic.

## 5. Conclusions

This scoping review identified several nursing interventions that empower families caring for older adults at risk of falling. The findings underscore the significance of therapeutic relationships, family involvement, personalized care, health education, multifactorial falls risk assessment, home modifications, referral practices, and seamless healthcare service transitions. The multifaceted nature of these elements emphasizes the need for a comprehensive and interconnected approach to fall prevention. The results of this scoping review underscore the significance of involving family caregivers in the care process as an effective means of addressing the risk and fear of falling in older adults. Establishing a care partnership, educating and empowering family caregivers, offering emotional support, and customizing interventions all fundamentally enhance older adults’ safety and quality of life, especially within their home environment. Therefore, nursing professionals need to recognize the value of involving family caregivers as critical partners in fall prevention and promoting the well-being of older adults.

## Figures and Tables

**Table 1:** Eligibility criteria.

Parameter	Inclusion Criteria	Exclusion Criteria
Population	Papers that focus on families caring for older adults.	Papers that do not focus on families that care for older adults.
Concept	Papers that explore interventions developed by nurses.	Papers addressing interventions outside the scope of nursing care.
Context	Papers related to the risk of falling in a home environment.	Papers unrelated to the risk of falling or home environment (e.g., hospital or long-term care facility).
Study design	All study designs, including experimental, observational, qualitative, mixed-methods, reviews, and other non-research formats such as editorials and viewpoints.	
Time frame	No time restrictions were imposed.	

**Table 2:** Data extraction and synthesis.

Author/Year/Title/Country	Study Design/Aim	Participants	Interventions	Outcomes
Pereira Oliveira et al., 2016 [31] Caregiver’s knowledge about prevention of falls in elderly Brazil	Exploratory descriptive study To analyze the caregiver’s knowledge about preventing falls in older adults.	20 participants	Health Education Multifactorial falls risk assessment	Caregivers possess some preventive attitudes and awareness of potential complications following a fall. However, their understanding remains superficial due to inadequate guidance on prevention.
Powell-Cope et al., 2017 [26] Teaching Family Caregivers to Assist Safely with Mobility United States of America	Literature review To teach family caregivers to assist safely with mobility.	Not applicable	Health Education Multifactorial falls risk assessment Referral	Not applicable
Powell-Cope et al., 2018 [27] Preventing Falls and Fall-Related Injuries at Home United States of America	Literature review To help nurses provide caregivers with the tools they need to manage their family member’s health care at home.	Not applicable	Health Education Multifactorial falls risk assessment	Not applicable
Kim et al., 2019 [24] Home modifications for older people with cognitive impairments: Mediation analysis of caregivers’ information needs and perceptions of fall risks United States of America	Cross-sectional correlational study To identify how many older people with cognitive impairments are living in modified homes; explore associated factors and examine the mediating effects that their caregivers’ information needs and perceptions of fall risk and other factors.	226 participants	Health Education Multifactorial falls risk assessment Health care consultant Family involvement Home modifications	Older adults with cognitive and functional impairments are more inclined to adapt their homes for the well-being of care recipients staying at home. Prioritizing caregivers’ information needs is crucial when contemplating home modifications to facilitate the care of older individuals with impaired activities of daily living.
Tan et al., 2021 [28] Dementia and Falls Management in Underserved Populations: The Cognition and Mobility Care Management Program United States of America	Exploratory observational study To describe the organization, implementation, and evaluation of an intervention for underserved and ethnically diverse older patients with dementia and fall risk.	272 participants	Health Education Multifactorial falls risk assessment Referral Personalized Care	A primary care–based screening and co-management program to identify and manage dementia and falls risk was well received, with high satisfaction and perceived benefit from patients and caregivers.
Montero-Odasso et al., 2022 [29] World guidelines for falls prevention and management for older adults: a global initiative Canada	Expert consensus To create evidence- and expert consensus-based fall prevention and management recommendations applicable to older adults for use by healthcare and other professionals.	Not applicable	Health Education Multifactorial falls risk assessment Referral Personalized Care	Not applicable
Killingback et al., 2020 [30] Transitions from healthcare to self-care: a qualitative study of falls service practitioners’ views on self-management United Kingdom	Exploratory descriptive study To understand the views of falls service practitioners regarding their role in supporting self-management of falls prevention; and a transition pathway from National Health Service exercise-based falls interventions to community-run exercise programs.	8 participants	Health Education Referral Personalized Care Health care consultant Therapeutic relationships Family involvement Transition between healthcare services	Some aspects of assisting older adults in self-management were deemed to be within or beyond the scope of fall service practitioners. Challenges in facilitating the transition to community-run programs included practitioner awareness and endorsement, patient acceptance, and patient suitability/program availability.
Yeni & Yilmaz, 2022 [32] Nurse-led home modification interventions for community-dwelling older adults with dementia and their impact on fall prevention Turkey	Quasi-experimental study To investigate the effects of nurse-led home modification interventions on the family members of home-dwelling older adults with dementia.	42 participants	Health Education Multifactorial falls risk assessment Home Modifications	Family-centered, nurse-led home-modification interventions can be effective in the prevention and reduction of falls in older adults with dementia.
Olson et al., 2022 [25] Promoting Safe Mobility United States of America	Literature review To explain strategies for partnering with caregivers to maximize older adults’ functional ability.	Not applicable	Health Education Multifactorial falls risk assessment Referral Personalized Care Therapeutic relationships	Not applicable

## Data Availability

The data presented in this study are available on request from the first author.
